# Optimization of Antifungal Extracts from *Ficus hirta* Fruits Using Response Surface Methodology and Antifungal Activity Tests

**DOI:** 10.3390/molecules201119648

**Published:** 2015-10-29

**Authors:** Chuying Chen, Chunpeng Wan, Xuan Peng, Yuhuan Chen, Ming Chen, Jinyin Chen

**Affiliations:** Jiangxi Key Laboratory for Postharvest Technology and Nondestructive Testing of Fruits & Vegetables, College of Agronomy, Jiangxi Agricultural University, Nanchang 330045, China; ccy0728@126.com (C.C.); lemonwan@126.com (C.W.); pengx1104@163.com (X.P.); chenyh5207@126.com (Y.C.); chenming50@126.com (M.C.)

**Keywords:** RSM, *Ficus hirta*, antifungal activity, optimization

## Abstract

The fruits of *Ficus hirta* (FH) display strong antifungal activity against *Penicillium italicum* and *Penicillium digitatum*. In order to optimize the extraction conditions of antifungal extracts from FH fruit, various extraction parameters, such as ethanol concentration, extraction time, solvent to solid ratio and temperature, were chosen to identify their effects on the diameters of inhibition zones (DIZs) against these two *Penicillium* molds. Response surface methodology (RSM) was applied to obtain the optimal combination of these parameters. Results showed that the optimal extraction parameters for maximum antifungal activity were: 90% (*v*/*v*) ethanol concentration, 65 min extraction time, 31 mL/g solvent to solid ratio and 51 °C temperature. Under the abovementioned extraction conditions, the experimental DIZs values obtained experimentally were 57.17 ± 0.75 and 39.33 ± 0.82 mm, which were very close to the values of 57.26 and 39.29 mm predicted by the model. Further, nine kinds of phytopathogens were tested *in vitro* to explore the antifungal activity of the FH extracts. It was found for the first time that the FH extracts showed significant inhibition on the growth of *P. italicum*, *A. citri*, *P. vexans*, *P. cytosporella* and *P. digitatum*.

## 1. Introduction

*Ficus hirta* Vahl. (Wuzhimaotao), a deciduous shrub of the family Moraceae, is widely distributed in southern China where it is used as a traditional plant resource both as a medicine and food by the Hakka people [1]. The fruits of *Ficus hirta* (FH) are used in Chinese folk medicine in the treatment of diuresis, difficult labor and puerperal pain, hepatitis and tumor [1,2]. It has many functional constituents, including flavonoids, coumarins and saponins, such as psoralen, bergapten, luteolin, apigenin, vitexin and 3,5,4′-trihydroxy-3,7-dimethoxyflavone [2,3,4,5]. Previous studies indicated that FH had good antioxidant [3], anti-inflammation [6], anti-radiation [7] and anti-tumour activities. Though considerable work has been done with regard to the components and pharmacological action of FH, very few reports are available with regard to the antimicrobial activity. Traditional Chinese herbal medicine is a rich and natural source of functional foods and pharmaceuticals. In the past decades, studies on the antimicrobial activities of medicinal plants have increased remarkably in number due to increased interest in their potential use as an important source of botanical fungicides [8,9,10,11].

Pathogen infection is an important factor that affects citrus fruit post-harvest physiology, disease resistance and metabolism. Blue and green citrus molds, caused by *Penicillium italicum* and *Penicillium digitatum*, respectively, are the most economically important postharvest diseases of citrus and cause heavy losses during storage, transportation and marketing, thus debasing the commodity value of the harvested fruits [12]. Both *Penicillium* molds may cause 60%–80% decay losses under ambient conditions [13], which lead to severe economic losses for exporting countries. At present, the primary means for controlling the above two *Penicillium* molds still relies mainly on the use of chemical fungicides. But excessive use of chemical fungicides may cause the development of fungicide-resistant pathogens [14]. There is another glaring problem with the increasing public concern over the potential impact of fungicide residues on human health and the environment, which is simply impossible to ignore. Biological control by natural botanical fungicides is considered to be a practical alternative to synthetic fungicide application.

The optimal extraction of active compounds from FH is an important step prior to the development of more antifungal extracts against pathogen in horticultural products. A number of techniques are available for the extraction of natural bioactive compounds from medicinal plants, such as steam distillation, water or solvent extraction, ultrasound-assisted extraction, microwave-assisted extraction, enzymatic extraction and supercritical fluid extraction [15,16,17]. Among these, ultrasound-assisted extraction (UAE) is an inexpensive, environmentally friendly, having a short extraction time and low solvent consumption efficient alternative to conventional extraction techniques. The peculiarity in extraction based on ultrasonic radiation pressure is mainly attributed to the effects of cavitation and mechanical vibration allowing greater penetration of solvent into the sample matrix and larger contact surface area between the solid and the solvent, and as a result, the target compounds more rapidly diffuses from the solid phase into the solvent [18].

To the best of our knowledge, there are no published reports on the use of UAE to extract antifungal compounds from FH fruits, and only limited information about the antifungal activity is known. Therefore, the aims of the current study were to optimize the process for extraction of antifungal extracts from FH fruits using response surface methodology (RSM) applying a central composite design (CCD) with three factors and five levels, to evaluate the antifungal potential of the obtained extracts.

## 2. Results and Discussion

### 2.1. The DIZs of FH Extracted with Different Extraction Solvents

**Figure 1:**
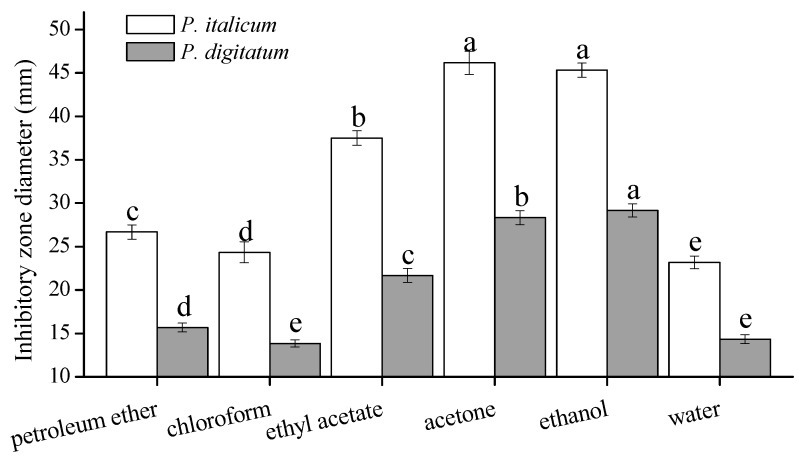
The effects of different extraction solvents on DIZs against *P. italicum* and *P. digitatum*. The vertical bars represent the standard deviation (*n* = 3). ^a–e^ Significant differences at *p* < 0.05 level. Extraction conditions under ultrasonic-assisted extraction: 60 min extraction time, 20:1 solvent to solid ratio and 20 °C temperature.

An extraction solvent system is generally selected according to the polarity of the objected and non-objected components, overall cost and safety [19]. In this paper, the effect of petroleum ether, chloroform, ethyl acetate, acetone, ethanol and distilled water extraction on the antifungal activity of FH were compared. Figure 1 shows the DIZs of FH extracted with different solvents. Significant difference in DIZs was observed among the various solvent extracts. Acetone extracts showed the maximum values of DIZ against *P. italicum* and had no significant difference as compared with ethanol extracts. Meanwhile, ethanol extracts contained the greatest value of DIZ against *P. digitatum*. In addition, ethanol is non-toxic, cheap, and widely applied. Therefore, ethanol was used as the extraction solvent in the following study.

### 2.2. Single Factor Experiments

**Figure 2:**
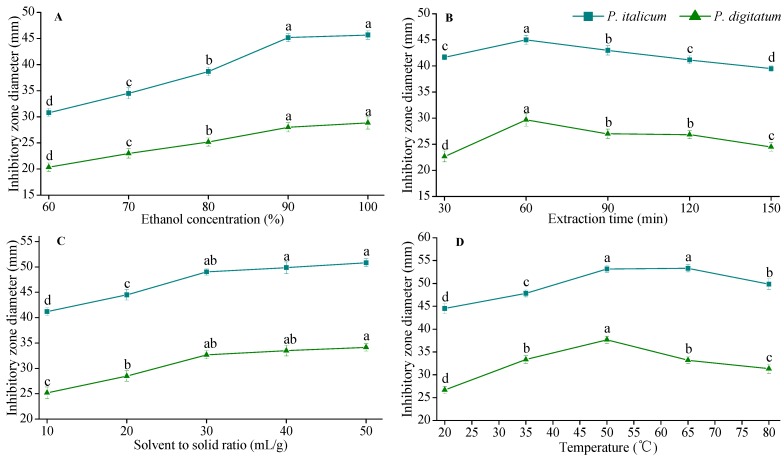
The effects of different extraction parameters on DIZs against *P. italicum* and *P. digitatum*. (**A**) Effect of ethanol concentration on DIZs, the other extraction conditions were 60 min extraction time, 20:1 solvent to solid ratio and 20 °C temperature; (**B**) Effect of extraction time on DIZs, the other extract condition were 90% ethanol, 20:1 solvent to solid ratio and 20 °C temperature; (**C**) Effect of solvent to solid ratio on DIZs, the other extract condition were 90% ethanol, 60 min time and 20 °C temperature; (**D**) Effect of temperature on DIZs, the other extraction conditions were 90% ethanol, 60 min extraction time and 30:1 solvent to solid ratio. (■) DIZs against *P. italicum*; (▲) DIZs against *P. digitatum*. ^a–d^ Significant differences at *p* < 0.05 level.

In this work, ethanol concentration, ultrasound extraction time, solvent to solid ratio and temperature were considered, and the results of those single factor experiments are presented in Figure 2.

#### 2.2.1. Effect of Ethanol Concentration on the DIZs of FH

The concentration of extraction solvent influences the DIZs of FH extracts. Generally, lower concentration of ethanol makes more of the larger polar antifungal compounds dissolve, whereas higher ethanol concentration is suitable for the extraction of less polar or non-polar ones [20]. The effect of the ethanol concentration on the DIZs against *P. italicum* and *P. digitatum* of FH is shown in Figure 2A. The DIZs were determined with different concentrations of ethanol (60%–100%, *v*/*v*) while other extraction parameters were left constant (60 min extraction time, 20 solvent to solid ratio and 20 °C temperature). The DIZs against *P. italicum* and *P. digitatum* increased significantly from 30.8 to 45.7 mm and 20.3 to 28.8 mm, when the concentration of ethanol increased from 60% to 100%. However, as the purity of ethanol reached the anhydrous state, the value of DIZs did not change significantly as compared with 90% ethanol. The ethanol concentration of 90%, therefore, was chosen as the optimal parameter.

#### 2.2.2. Effect of Extraction Time on the DIZs of FH

Extraction time is a distinctly important factor that would significantly influence the DIZs of FH. With too short an extraction time the antifungally active compounds in FH fruits cannot be completely extracted. Too long an extraction time will cause high energy costs [21]. The extraction time was set to 30, 60, 90, 120 and 150 min, whereas other extration parameters were set as follows: 90% ethanol, 20:1 solvent to solid ratio and a temperature of 20 °C. According to Figure 2B, when the extraction time varied from 30 min to 60 min, the DIZs against *P. italicum* and *P. digitatum* were significantly increased. However, there was a significant decrease when the time was extended beyond 60 min. The results indicated that a longer extraction time has a positive effect on the antifungal efficacy of FH as long as it does not exceed 60 min. Meanwhile, too long an extraction time may lead to the degradation of the antifungal compounds of FH. Therefore, 60 min was chosen as the favorable extraction time.

#### 2.2.3. Effect of Solvent to Solid Ratio on the DIZs of FH

The effect of solvent to solid ratio on the DIZs of FH is shown in Figure 2C. Extraction was carried out at different solvent to solid ratios (10–50 mL/g, *v*/*w*) when other extration parameters were set as follows: 90% ethanol, 60 min extraction time and 20 °C temperature. The DIZs against *P. italicum* and *P. digitatum* increased significantly from 41.2 to 50.8 mm and 25.2 to 34.1 mm as the solvent to solid ratio increased within the range of 10 to 30 mL/g (*v*/*w*), due to the increase of the driving force for the mass transfer of the FH fruit components. This indicated that more solvent contributed to sufficient dissolution of target antifungal compounds as the driving force of molecules was supposed to be higher at a higher solvent to solid ratio [22]. However, the DIZs no longer significantly changed when the solvent to solid continued to increase. Thus, 30 mL/g was selected as the most favorable solvent to solid ratio.

#### 2.2.4. Effect of Temperature on the DIZs of FH

Figure 2D shows the effects of temperature on the DIZs of FH extracts. Extraction was carried out at 20, 35, 50, 65 and 80 °C, while other extraction parameters were set as follows: 90% ethanol, 60 min extraction time and 30:1 solvent to solid ratio. The DIZs significantly increased when the temperature increased from 20 to 50 °C, and reached the maximum values (53.2 and 37.7 mm). Higher than 70 °C, the values of DIZs decreased significantly. The reason might be that the solubility of the antifungal active compounds increases with the rise in extraction temperature; however, when the extraction temperature continued to rise, a decline in DIZs was observed due to the heat sensitivity of some antifungal active compounds reduced [23,24]. Therefore, 50 °C was chosen as the optimal extraction temperature based on the experimental data.

### 2.3. Statistical Analysis and the Model Fitting

To maximize DIZs against *P. italicum* and *P. digitatum*, extraction conditions were optimized using response surface methodology. A CCD with five-levels-three-factor was used for three extraction variables, such as extraction time, solvent to solid ratio and temperature (Table 1). To choose the best model agreeing with the data, the analysis of variance and goodness-of-fit by calculating F and *p*-value was summarized (Table 2 and Table 3). The DIZs against *P. italicum* and *P. digitatum* ranged from 46.6 to 57.4 mm and 31.8 to 39.5 mm. The maximum DIZs (57.4 and 39.5 mm) were recorded for an extraction time of 60 min, solvent to solid ratio of 30 mL/g and temperature of 50 °C. There is an empirical relationship between the response variable (DIZs against *P. italicum* and *P. digitatum*) and the test variable under consideration.

As Table 2 shows, by applying multiple regression analysis to the experiment data, the experimental results of the CCD were fitted with a second-order polynomial regression equations (Equation (1)). The Equation (2) was fitted to the DIZs against *P. digitatum* was presented, as follows:

(1) $${\text{Y}}_{1}=57.07+1.31{\text{X}}_{1}+0.27{\text{X}}_{2}+0.58{\text{X}}_{3}+2.54{\text{X}}_{1}^{2}-2.10{\text{X}}_{2}^{2}-2.33{\text{X}}_{3}^{2}-0.39{\text{X}}_{1}{\text{X}}_{2}-0.99{\text{X}}_{1}{\text{X}}_{3}-0.31{\text{X}}_{2}{\text{X}}_{3}$$

(2) $${\text{Y}}_{2}=39.23+0.34{\text{X}}_{1}+0.54{\text{X}}_{2}+0.23{\text{X}}_{3}-1.81{\text{X}}_{1}^{2}-1.90{\text{X}}_{2}^{2}-2.13{\text{X}}_{3}^{2}+0.40{\text{X}}_{1}{\text{X}}_{2}-0.87{\text{X}}_{1}{\text{X}}_{3}+0.25{\text{X}}_{2}{\text{X}}_{3}$$

**Table 1:** A central composite design for independent variables and their responses.

Standard Order	Run Order	Coded Level of Fermentation Condition			DIZs (mm)	
		X_1_ (min)	X_2_ (mL/g)	X_3_ (°C)	Y_1_	Y_2_
1	14	−1 (30)	−1 (20)	−1 (35)	46.6	32.2
2	3	1 (90)	−1 (40)	−1 (35)	51.6	33.8
3	12	−1 (30)	1 (40)	−1 (35)	48.0	31.8
4	4	1 (90)	1 (40)	−1 (35)	52.2	35.4
5	6	−1 (30)	−1 (20)	1 (65)	49.7	33.5
6	20	1 (90)	−1 (20)	1 (65)	51.5	32.0
7	18	−1 (30)	1 (40)	1 (65)	50.6	34.5
8	1	1 (90)	1 (40)	1 (65)	50.1	34.2
9	13	−1.68 (9.5)	0 (30)	0 (50)	47.8	33.7
10	17	1.68 (110.5)	0 (30)	0 (50)	52.2	34.4
11	9	0 (60)	−1.68 (13.2)	0 (50)	50.6	32.9
12	7	0 (60)	1.68 (46.8)	0 (50)	51.9	34.7
13	2	0 (60)	0 (30)	−1.68 (24.8)	49.3	32.5
14	16	0 (60)	0 (30)	1.68 (75.2)	51.9	33.8
15	5	0 (60)	0 (30)	0 (50)	57.3	39.2
16	15	0 (60)	0 (30)	0 (50)	57.1	39.5
17	10	0 (60)	0 (30)	0 (50)	56.9	39.1
18	8	0 (60)	0 (30)	0 (50)	57.4	39.0
19	11	0 (60)	0 (30)	0 (50)	57.0	39.2
20	19	0 (60)	0 (30)	0 (50)	56.7	39.4

**Table 2:** Results of regression analysis and corresponding *F* and *p-*value of second-order model polynomial regression equation for DIZs against *P. italicum* and *P. digitatum*.

Source	Coefficient	Standard Error	*F*-Value	*p*-Value
DIZs against *P. italicum*				
Intercept	57.07	0.143	281.46	<0.0001
X_1_	1.31	0.095	190.5	<0.0001
X_2_	0.27	0.095	8.08	0.0175
X_3_	0.58	0.095	36.85	0.0001
${\text{X}}_{1}^{2}$	−2.54	0.092	753.8	<0.0001
${\text{X}}_{2}^{2}$	−2.10	0.092	514.14	<0.0001
${\text{X}}_{3}^{2}$	−2.33	0.092	633.06	<0.0001
X_1_X_2_	−0.39	0.124	9.75	0.0108
X_1_X_3_	−0.99	0.124	63.34	<0.0001
X_2_X_3_	−0.31	0.124	6.34	0.0305
DIZs against *P. digitatum*				
Intercept	39.23	0.105	383.18	<0.0001
X_1_	0.34	0.07	23.04	0.0007
X_2_	0.54	0.07	60.65	<0.0001
X_3_	0.23	0.07	11.16	0.0075
${\text{X}}_{1}^{2}$	−1.81	0.068	710.76	<0.0001
${\text{X}}_{2}^{2}$	−1.90	0.068	781.78	<0.0001
${\text{X}}_{3}^{2}$	−2.13	0.068	982.25	<0.0001
X_1_X_2_	0.40	0.091	19.22	0.0014
X_1_X_3_	−0.87	0.091	91.97	<0.0001
X_2_X_3_	0.25	0.091	7.51	0.0208

**Table 3:** ANOVA for the effects of extraction time (X_1_), solvent to solid ratio (X_2_) and temperature (X_3_) on DIZs against *P. italicum* and *P. digitatum*.

Source	Sum of Squares	Df	Mean Square	*F*-Value	*p*-Value
DIZs against *P. italicum*					
Model	234.50	9	26.06	211.56	<0.0001
Residual	1.23	10	0.12		
Lack of Fit	0.90	5	0.18	2.69	0.1503
Pure Error	0.33	5	0.067		
Cor Total	235.73	19			
R^2^ = 0.9948, Adj. R^2^ = 0.9901, Pred. R^2^ = 0.9672, CV = 0.67					
DIZs against *P. italicum*					
Model	151.94	9	16.88	253.50	< 0.0001
Residual	0.67	10	0.07		
Lack of Fit	0.49	5	0.10	2.84	0.1381
Pure Error	0.17	5	0.03		
Cor Total	152.61	19			
R^2^ = 0.9956, Adj. R^2^ = 0.9917, Pred. R^2^ = 0.9730, CV = 0.73					

The significance of each coefficient was determined using *F*-test and *p*-values as shown in Table 2. ANOVA analysis was used for checking the signficance and suitability of the model, and a statistical summary is shown in Table 3. It was considered as signficant if the *F*-value becomes greater and the *p*-value becomes smaller. Lack of fit was also determined to check the quality of the model. According to Table 3, the ANOVA for the response surface quadratic regression model showed that the model was highly significant (*p* < 0.0001) with a very high *F*-value (211.56 and 253.50).

**Figure 3:**
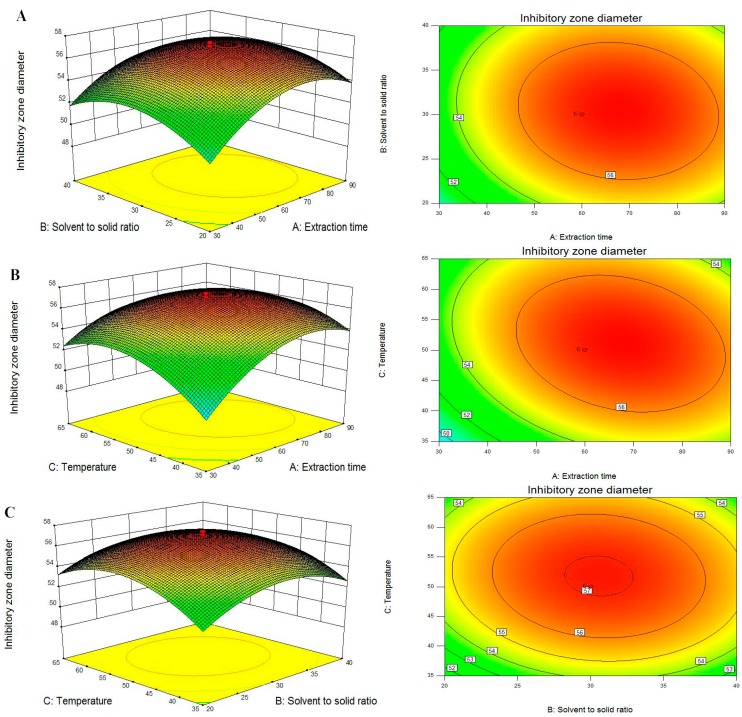
Response surface plot and contour plot showing DIZs against *P. italicum* of extraction time and solvent to solid ratio (**A**); extraction time and temperature (**B**) and solvent to solid ratio and temperature (**C**).

**Figure 4:**
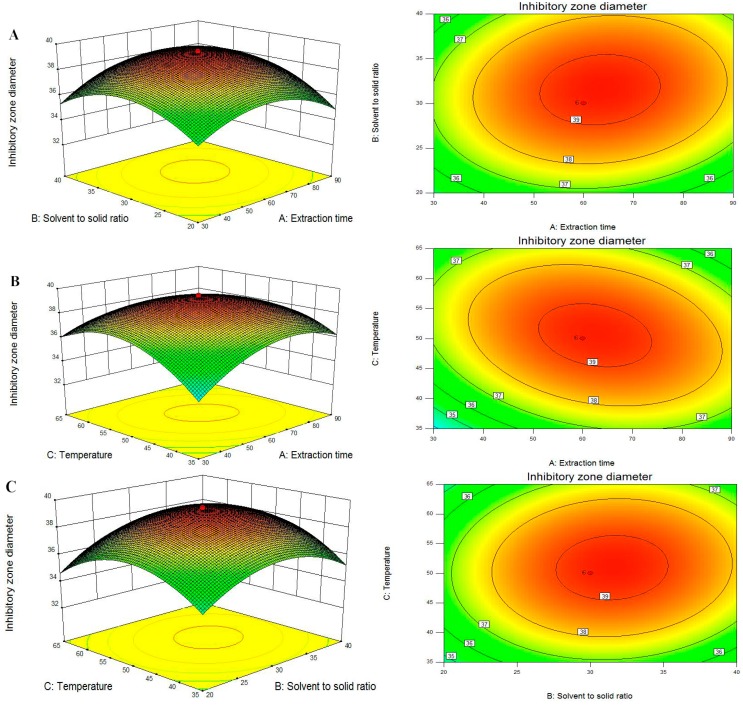
Response surface plot and contour plot showing DIZs against *P. digitatum* of extraction time and solvent to solid ratio (**A**); extraction time and temperature (**B**) and solvent to solid ratio and temperature (**C**).

For the model fitting, the coefficient of R^2^ (coefficient of determination) and Adj. R^2^ (adjustable R^2^) as well as Pred. R^2^ (predictable R^2^) were checked. The R^2^ value indicates how well of the model fits the experimental data, and the closer R^2^ to one, the more significant the model and the better it predicts the response. The values of R^2^ were 0.9948 and 0.9956, indicating that there is a good correlation between the experimental and predicted values and only 0.52% and 0.44% of the total variations (DIZs against *P. italicum* and *P. digitatum*) was not explained by the response model. In addition, the Adj. R^2^ and Pred. R^2^ are also essential parameters to check adequate precision. The Adj. R^2^ is a modification of R^2^ and attempts to yield a more honest value to estimate R^2^, the Pred. R^2^ is used to indicate how well the model predicts response for new observations. In this case, the values of the Adj. R^2^ were 0.9901 and 0.9917, which was also satisfactory to confirm that the model was highly significant. Meanwhile, the values of Pred. R^2^ (0.9672 and 0.9730) were in reasonable agreement with the values of Adj. R^2^. The *F*-values for the lack of fit were 0.1503 and 0.1381, which is insignificant relative to the pure errors, thereby confirming the validity of the model. At the same time, the very low values (0.67 and 0.73) of coefficient of variation (CV) clearly declared a very high degree of precision and a good deal of reliability of the experimental values.The three-dimensional (3D) response surfaces and two-dimensional (2D) contour plots are the graphical representations of regression equation, which provide a method to visually display the relationship between responses and experimental levels of each independent variable and the type of interactions between two test variables [25]. From the 3D response surfaces and 2D contours shown in Figure 3 and Figure 4, each figure shows the effects of the two independent variables and their mutual interactions on the DIZs against *P. italicum* and *P. digitatum* while the third one was kept at zero level.

Figure 3A and Figure 4A are the 3D plots and contour plots showing the effect of extraction time and solvent to solid ratio on the response at a fixed temperature. From these figures, it can be clearly seen that DIZs against *P. italicum* and *P. digitatum* increased with increasing extraction time and solvent to solid ratio, but further increasing the solvent to solid ratio would not significantly increase the DIZs. The results are in accordance with single factor test and the ANOVA analysis (Table 2). The results are also in agreement with reports by the authors reporting the extraction of continentalic acid from the root of *Aralia continentalis* [26].

Figure 3B and Figure 4B show the 3D plots and contour plots at varying extraction time and temperature. As shown in these figures, the DIZs against *P. italicum* and *P. digitatum* increased with increasing extraction time and temperature of the raw material at the initial stage. Any additional increase in the extraction temperature had only a slight effect on DIZs. A similar trend has been reported for *Andrographis paniculata* diterpenoids [27] and *Mangifera indica* L. mangiferin [28].

The 3D plots and the contour plots based on the independent variables solvent to solid ratio and extraction temperature are shown in Figure 3C and Figure 4C, while the extraction time was kept at zero level (60 min). The variation trends of DIZs against *P. italicum* and *P. digitatum* reached a maximum, and beyond this point, additional increases in the solvent to solid ratio and extraction temperature did not improve the DIZs.

To sum up, from the 3D response surfaces and contour plots, the optimum extraction parameters within the experimental ranges were extraction time 67.25 and 62.98 min, solvent to solid ratio 30.38 and 31.37 mL/g, temperature 51.06 and 50.66 °C, respectively. Under these conditions, the maximum predicted DIZs against *P. italicum* and *P. digitatum* were 57.26 and 39.29 mm.

### 2.4. Optimization Responses and Validation of the Model

The numerical optimisation of extraction parameters were carried out using Design-Expert statistical software based on the initial experimental results. In order to validate the adequacy of the model equations (Equations (2) and (3)) and obtain the maximum value of DIZs of antifungal extracts against *P. italicum* and *P. digitatum* from the FH fruits, the validation test was carried out according to the two predicted and optimal extraction conditions. Extraction conditions were adjusted: 90% (*v*/*v*) ethanol concentration, 65 min extraction time, 31 mL/g solvent to solid ratio and 51 °C temperature. The result showed that the DIZs against *P. italicum* and *P. digitatum* were 57.17 and 39.33 mm, indicating that the experimental values were not only in close agreement with the predicted values but also higher than any single-factor experiment, and the deviation was found to be insignificant (Table 4). Therefore, the model was considered to be accurate and feasible for predicting the DIZs of antifungal extracts from FH fruits.

**Table 4:** Response values under optimal conditions of ethanol concentration (90%), extraction time (65 min), solvent to solid ratio (31 mL/g) and temperature (51 °C).

Responses	Predicted Value	Experimental Value
DIZs against *P. italicum* (mm)	57.22	57.17 ± 0.75
DIZs against *P.digitatum* (mm)	39.28	39.33 ± 0.82

### 2.5. Antifungal Spectrum of FH Extracts

Under the optimal extraction conditions, the FH extracts showed potent *in vitro* antifungal effects against the nine phytopathogens, measured as EC_50_ values. As shown in Table 5, the EC_50_ values of FH extracts against the tested phytopathogens were found in the range of 5–40 mg/mL. The main reason is that the bioactivities varied depending on the phytopathogens tested, leading to different values of its EC_50_. The FH extracts exhibited the more efficient activity against *P. italicum*, *A. citri*, *P. vexans*, *P. cytosporella* and *P. digitatum*, and had weaker activity against *B. cinerea*, *B. dothidea* and *A. alternate*. In this assay, *P. italicum*, *P. digitatum*, *A. citri*, *P. cytosporella* and *G. citri-aurantii*, which are the causal agents of postharvest citrus blue mold, green mold, black rot, stem-end rot and sour rot, were found to be extremely susceptible phytopathogens to the FH extracts, with EC_50_ values of 5.04, 7.95, 5.15, 7.01 and 13.06 mg/mL. In addition, *B. cinerea*, *B. dothidea* and *A. alternate* showed less susceptibility to the FH extracts with high EC_50_ values. FH extract showed strong antifungal activity against the nine phytopathogens under *in vitro* conditions and had a broad antifungal spectrum compared with the results of Askarne and Talibi, who have previously reported that 100 mg/mL crude extracts of several Moroccan plants, such as *Trichodesma calcaratum* Coss. ex Batt., *Ruta chalepensis* L. and *Cistus crispus* L. can effectively inhibited the mycelial growth at 47.4% to 57.7% of citrus blue mold, and also found that 100 mg/mL aqueous extracts from *Cistus crispus* L. and *Trichodesma calcaratum* Coss. ex Batt. totally inhibited 44.4% and 52.5% of citrus sour rot [29,30].

**Table 5:** Toxicities of the FH extracts against phytopathogens.

Species	Toxicity Regression Equation	R^2^	EC_50_ (mg/mL)
*P. italicum*	Y = 4.1301 + 1.5571X	0.9963	5.04
*A. citri*	Y = 4.4794 + 0.7442X	0.9894	5.15
*P. vexans*	Y = 3.3269 + 1.8864X	0.9940	6.98
*P. cytosporella*	Y = 3.9207 + 1.4206X	0.9982	7.01
*P. digitatum*	Y = 3.3381 + 1.7307X	0.9978	7.95
*G. citri-aurantii*	Y = 3.2278 + 1.2738X	0.9905	13.06
*B. cinerea*	Y = 3.4513 + 1.2602X	0.9884	22.43
*B. dothidea*	Y = 3.4036 + 1.2048X	0.9957	25.80
*A. alternate*	Y = 3.1294 + 1.1674X	0.9914	39.21

X denotes the concentration of FH extracts (natural logarithm). Y denotes the rate of mortality. R^2^ denotes the coefficient of determination.

## 3. Experimental Section

### 3.1. Reagents and Plant Materials

The fruits of *Ficus Hirta* (origin: Guangdong Province, China) were purchased from the Huafeng herbs store in Zhangshu (Jiangxi Province, China). The samples were powdered in a grinder and sieved (less than 20 mesh) after drying below 30 °C for 15 h. The fine powder sample was stored in hermetically sealed bags at room temperature (about 25 °C). Ethanol and glucose were of analytical grade. The agar powder was purchased from Solarbio (Beijing, China).

### 3.2. Pathogens

*Penicillium italicum* and *Penicillium digitatum* were provided by the Jiangxi Key Laboratory for Postharvest Technology and Nondestructive Testing of Fruits & Vegetables (Nanchang, China). All the test strains were preserved on potato dextrose agar (PDA) at 4 ± 0.5 °C. The spores’ concentrations were determined with the aid of a hematocyte counter and adjusted to 10^5^–10^6^ CFU/mL with sterile distilled water.

The other seven phytopathogens were used: *Geotrichum citri-aurantii*, *Alternaria citri*, *Phomopsis cytosporella*, *Botryosphaeria dothidea*, *Botrytis cinerea*, *Phomopsis vexans*, and *Alternaria alternate*. They were provided by the key laboratory for plant pathology of Jiangxi Agricultural University (Nanchang, China) and incubated on PDA at 27 ± 1 °C.

### 3.3. Selection of Extraction Solvent

FH powder (5.0 g) was extracted with 100 mL each of selected solvents of increasing polarity: petroleum ether, chloroform, ethyl acetate, acetone, ethanol and distilled water, respectively, in a conical flask with a stopper (250 mL) and kept for ultrasonic-assisted extraction at room temperature. After 30 min, all of the filtrate of organic extracts were concentrated under reduced pressure below 40 °C using a rotary evaporator (Buchi Rotavapor R-3, Flawil, Switzerland). The filtrate of aqueous extract obtained was quickly frozen at −40 °C and dried by a Labconco FDU-1200 freeze dryer (Tokoy Rikakikai Co. Ltd., Tokoy city, Japan) for 48 h. The remaining solution of 1 g/mL (raw herb/solvent: *w*/*v*) was referred to as the organic extracts and the dried aqueous extract was dissolve with 5 mL of distilled water. All of the crude extracts were stored at 4 °C for further analysis.

### 3.4. Determination of DIZ

The antifungal activities of FH extracts against the two *Penicillium* molds were evaluated by the disk diffusion method [31]. Petri dishes (diameter, 9 cm) were prepared with PDA and surface inoculated with 2% of spore suspensions (10^5^–10^6^ CFU/mL) in sterile saline solution. Sterile Oxford cup (diameter, 8 mm) were impregnated with 200 μL of each extract. The DIZs around the Oxford cups were evaluated after 48 h of culture at 27 ± 1 °C in the darkness. For each mold, three replicate trials were conducted against each extract.

### 3.5. Experimental Design

#### 3.5.1. Single Factor Experiments

A screening study was carried out to determine appropriate ranges of independent variables, such as ethanol concentration, ultrasound extraction time, solvent to solid ratio and temperature to be applied in design of multifactor experiments. Firstly, the effect of ethanol concentration on the extraction was investigated. The dried powder (5.0 g) was added ethanol of concentrations of 60%, 70%, 80%, 90%, 100% for a fixed ultrasound-assisted extraction time of 60 min, solvent to solid ratio of 20 mL/g (20 mL of ethanol to 1 g powder) and extraction temperature of 20 °C. Secondly, we performed the influence of the ultrasound extraction time on DIZs against the two *Penicillium* molds. 100 mL of 90% ethanol was added, and extraction performed for different times of 30, 60, 90, 120, 150 min at 20 °C. Thirdly, the solvent to solid ratio 90% ethanol was evaluated at different ratios from 10 to 50 at a fixed extraction time of 60 min and extraction temperature of 20 °C. Lastly, the impact of extraction temperature on DIZs in the range from 20 to 80 °C was studied. 150 mL of 90% ethanol was added and the extraction performed at different temperatures (20–80 °C) for 60 min. The filtrate of each treatment was concentrated by means of vacuum distillation at 45 °C using a Buchi rotary evaporator. The remaining solution of 1 g/mL was referred to as the crude extracts and stored at 4 °C for further analysis.

#### 3.5.2. Optimization of Extraction Conditions

On the basis of the single factor experiment results, we confirmed ethanol concentration at 90%, and selected extraction time (min, X_1_), solvent to solid ratio (mL/g, X_2_) and temperature (°C, X_3_) as independent variables that should be optimised for the extraction (Table 6). In the study, the experiments were performed following the Central Composite Design (CCD). The levels of the experimental factors for the CCD are shown in Table 1. The complete design was carried out in random order and consisted of 20 combinations including five replicates of central point (Table 1). The data from CCD were analysed by multiple regression to fit the following quadratic polynomial model (see Equation (3)).

(3) $$\text{Y}={\text{γ}}_{0}+\sum_{\text{i}=1}^{3}\alpha_{\text{i}}{\text{X}}_{i}+\sum_{\text{i}=1}^{3}\alpha_{\text{i}i}{\text{X}}_{i}^{2}+\sum_{\text{i}=1}^{2}\sum_{\text{i}=i+1}^{3}\alpha_{\text{i}j}X_{\text{i}}X_{\text{j}}$$

where Y is the predicted response, γ_0_ is the constant (interception), α_i_, α_ii_ and α_ij_ are the linear, quadratic and cross product coefficients for ultrasonic time, liquid–solid ratio and temperature, respectively. Accordingly, X_i_ and X_j_ are independent variables, respectively. The response surface design were used to analyze the experimental results with Design-Expert 8.0.6 software (Trial version, State-Ease Inc., Minneapolis, MN, USA). *p*-Values < 0.05 were considered to be statistically significant. All experiments were conducted in triplicate.

### 3.6. In-Vitro Bioassay Assay

The antifungal activities of the FH extracts against nine kinds of plant pathogens were examined by the method of Lee [32]. The FH extracts were dissolved in sterile distilled water with 0.1% Tween 80, and then added to the sterile culture medium (PDA) at the specified concentrations. The mixed media were poured into plastic Petri dishes (90 mm). Following thorough coagulating, the agar-mycelial plugs (6 mm) infected with fungi were incubated at the centre of the dish. The Petri dishes were sealed with parafilm and incubated in the dark. Colony growth diameters were measured after the fungal growth in the control treatment had completely covered the Petri dishes. All treatments were tested in four replicates, and plates without any additives were used as controls. The EC_50_ values and P-test were carried out by SPSS 17.0 software. The fungi toxicity of FH extracts was expressed as percentage inhibition of mycelial growth (IMG, %) using the following formula: IMG (%) = (dc − dt)/(dc − 6) × 100, where dc and dt were the averages from four replicates of mycelium diameters (mm) of the control and the treatment, respectively.

**Table 6:** Range of extraction condition variables at different levels for the central composite design.

Independent Variables	Parameter	Coded Levels				
		−1.68	−1	0	1	1.68
Extraction time (min)	X_1_	9.5	30	60	90	110.5
Solvent to solid ratio (mL/g)	X_2_	13.2	20	30	40	46.8
Temperature (°C)	X_3_	24.8	35	50	65	75.2

## 4. Conclusions

In the present paper, the antifungal extracts from *Ficus hirta* ftuits were obtained with a three-factor, five-level CCD based on the RSM. The method proved to be very useful and reliable tool for preparation and optimization of antifungal extracts of FH extraction. The statistical analysis showed that ethanol concentration of 90%, extraction time of 65 min, solvent to solid ratio of 31 mL/g, and temperature of 51 °C were the best conditions to obtain antifungal extracts from FH. Under the optimal extraction conditions, the DIZs obtained experimentally were 57.17 ± 0.75 and 39.33 ± 0.82 mm, which were very close to the values predicted by the model, namely 57.26 and 39.29 mm. In addition, the FH extracts were separately assayed for inhibition of nine kinds of phytopathogens *in vitro*, and showed significant inhibition on the growth of *P. italicum*, *A. citri*, *P. vexans*, *P. cytosporella* and *P. digitatum*. According to the results, it can be concluded that the FH extracts have a broad antifungal spectrum.
